# Left Ventricular Diastolic Dysfunction Is Associated with Poor Functional Outcomes after Endovascular Thrombectomy

**DOI:** 10.3390/jcdd11030087

**Published:** 2024-03-05

**Authors:** Tony Y. W. Li, Emma M. S. Toh, Ying Ying Koh, Aloysius S. T. Leow, Bernard P. L. Chan, Hock-Luen Teoh, Raymond C. S. Seet, Anil Gopinathan, Cunli Yang, Vijay K. Sharma, Leonard L. L. Yeo, Mark Y. Chan, William K. F. Kong, Kian-Keong Poh, Benjamin Y. Q. Tan, Ching-Hui Sia

**Affiliations:** 1 Department of Cardiology, National University Heart Centre Singapore, 1E Kent Ridge Road, NUHS Tower Block Level 9, Singapore 119228, Singapore; tony.li@mohh.com.sg (T.Y.W.L.); mark_chan@nuhs.edu.sg (M.Y.C.); william_kong@nuhs.edu.sg (W.K.F.K.); kian_keong_poh@nuhs.edu.sg (K.-K.P.); 2 Department of Medicine, Yong Loo Lin School of Medicine, National University of Singapore, Singapore 119228, Singapore; emma.toh@mohh.com.sg (E.M.S.T.); yingying.koh@mohh.com.sg (Y.Y.K.); aloysius.leow@mohh.com.sg (A.S.T.L.); bernard_chan@nuhs.edu.sg (B.P.L.C.); hock_luen_teoh@nuhs.edu.sg (H.-L.T.); raymond_cs_seet@nuhs.edu.sg (R.C.S.S.); vijay_kumar_sharma@nuhs.edu.sg (V.K.S.); leonard_ll_yeo@nuhs.edu.sg (L.L.L.Y.); benjamin_yq_tan@nuhs.edu.sg (B.Y.Q.T.); 3 Division of Neurology, Department of Medicine, National University Health System, Singapore 119228, Singapore; 4 Division of Interventional Radiology, Department of Diagnostic Imaging, National University Health System, Singapore 119228, Singapore; anil_gopinathan@nuhs.edu.sg (A.G.); cunli_yang@nuhs.edu.sg (C.Y.)

**Keywords:** atrial fibrillation, ischaemic stroke, cerebrovascular disease, stroke, embolism, diastolic dysfunction

## Abstract

Introduction: With the advent of endovascular thrombectomy (ET), patients with acute ischaemic strokes (AIS) with large vessel occlusion (LVO) have seen vast improvements in treatment outcomes. Left ventricular diastolic dysfunction (LVDD) has been shown to herald poorer prognosis in conditions such as myocardial infarction. However, whether LVDD is related to functional recovery and outcomes in ischaemic stroke remains unclear. We studied LVDD for possible relation with clinical outcomes in patients with LVO AIS who underwent ET. Methods: We studied a retrospective cohort of 261 LVO AIS patients who had undergone ET at a single comprehensive stroke centre and correlated LVDD to short-term mortality (in-hospital death) as well as good functional recovery defined as modified Rankin Scale of 0–2 at 3 months. Results: The study population had a mean age of 65-years-old and were predominantly male (54.8%). All of the patients underwent ET with 206 (78.9%) achieving successful reperfusion. Despite this, 25 (9.6%) patients demised during the hospital admission and 149 (57.1%) did not have good function recovery at 3 months. LVDD was present in 82 (31.4%) patients and this finding indicated poorer outcomes in terms of functional recovery at 3 months (OR 2.18, 95% CI 1.04–4.54, *p* = 0.038) but was not associated with increased in-hospital mortality (OR 2.18, 95% CI 0.60–7.99, *p* = 0.240) after adjusting for various confounders. Conclusion: In addition to conventional echocardiographic indices such as left ventricular ejection fraction, LVDD may portend poorer outcomes after ET, and this relationship should be investigated further.

## 1. Introduction

The treatment of acute ischaemic stroke (AIS) especially those with large vessel occlusion (LVO) has been revolutionized with the advent of endovascular thrombectomy (ET), which offers the potential for effective reperfusion [1,2]. In selected patients, this approach reduces mortality and improves functional outcomes [3]. However, such LVO strokes continue to herald high mortality and morbidity with less than half achieving good functional outcomes despite reperfusion with ET [4].

Cardiovascular diseases including ischaemic heart disease are common in stroke patients probably due to similar underlying comorbidities and a shared pathophysiology [5]. Left ventricular systolic dysfunction (LVSD) is often seen in patients with LVO AIS and such impaired cardiac function increases the risk of poor neurologic and functional outcomes after ET [6,7,8]. However, the role of left ventricular diastolic dysfunction (LVDD) remains unclear. LVDD and elevated left ventricular filling pressures are related to adverse outcomes in patients with acute myocardial infarction, heart failure, as well as stroke [9,10,11,12]. Among stroke patients, LVDD and markers of elevated filling pressures are considered as possible markers for identifying occult cardioembolism in patients with cryptogenic stroke and have been shown to be associated with atrial fibrillation, recurrent stroke and poor outcome [13,14].

In this study, we sought to evaluate the relationship between LVDD and clinical outcomes in our cohort of LVO stroke patients who underwent ET.

## 2. Methodology

### 2.1. Study Design

This study evaluated a retrospective cohort of LVO AIS patients from a single comprehensive stroke centre who were treated with ET between 2013 and 2019 and underwent a valid transthoracic echocardiography (TTE). Prior to ET, all patients underwent baseline neurovascular imaging with computed tomography (CT) and computed tomography angiography. Patients were administered bridging intravenous thrombolysis if they presented within 4.5 h of symptom-onset and had no contraindications. LVO was defined as occlusions seen in the basilar artery (BA), internal carotid artery (ICA), proximal segments of the middle cerebral artery (M1 and/or M2) or cervical ICA. Patients were considered eligible for ET if the procedure was initiated within 6 h of the time of stroke onset and had an angiographically confirmed occlusion. Exceptional cases presenting later than 6 h were performed in accordance with AHA/ASA guidelines [15,16]. The National Institutes of Health Stroke Scale (NIHSS) score on arrival and Alberta Stroke Program Early CT Score (ASPECTS) was routinely used to assess the severity of stroke. The definitions of the NIHSS scale, ASPECTS score, modified Rankin Scale and TOAST classification are available in the Supplementary Materials. Patients were excluded from ET when pre-treatment imaging revealed extensive ischaemic changes in the brain (ASPECTS < 6), or if the stroke was considered mild, based on an admission NIHSS score of 3 or less. There was no upper limit on either the age of the patient or admission NIHSS score for inclusion.

Clinical monitoring was performed in intensive care or a high-dependency unit setting during the initial acute episode. All patients were managed according to international guidelines for the management of acute ischaemic stroke. ET employed either a stent-retriever, direct aspiration catheter, or combined stent-retriever and aspiration via intermediate catheter approach from the start of the procedure. Follow-up CT scan was performed at around 24 h after the initial treatment. Patients who required intracranial stenting and intra-arterial thrombolysis were excluded from this study. Additionally, patients with a premorbid modified Rankin scale (mRS) ≥2 were excluded from this study.

Baseline data collected for all patients included their demographics as well as comorbidities such as hypertension, diabetes mellitus (DM), dyslipidaemia (HLD), ischaemic heart disease (IHD), previous stroke or transient ischaemic attack (TIA), and atrial fibrillation (AF). NIHSS score and ASPECTS on arrival were recorded in addition to the details of the ET procedure performed. Expanded Treatment in Cerebral Infarction (eTICI) score was used to assess for reperfusion, where a eTICI grade of 2b-3 was considered successful reperfusion [17]. All patients were followed for their progress and various outcome measures were measured including the modified Rankin Scale (mRS) at 3 months, in-hospital mortality, reperfusion and symptomatic intracerebral haemorrhage (ICH). Appearance of new parenchymal haemorrhage on the follow up CT scan with an increase on NIHSS by ≥4 points constituted a symptomatic ICH [18]. The mRS score at 3 months was used as the measure of recovery and a score of 0–2 represented functional independence [19].

### 2.2. Echocardiographic Analysis

For all patients in this study, a transthoracic echocardiogram (TTE) was performed within 6 months of the AIS episode. Standard measurements of various echocardiographic and Doppler parameters were made in accordance with American Society of Echocardiography guidelines [20]. In accordance to international guidelines, the biplane Simpson’s method was used to assess end-diastolic volume (EDV) and end-systolic volume (ESV) as well as left ventricular ejection fraction (LVEF). Pulsed-wave Doppler was also applied in the apical 4-chamber view to assess mitral inflow including parameters such as early diastolic (E-wave) velocity and deceleration time. In the similar view, spectral tissue Doppler imaging was also performed and early diastolic (e’) was derived from the basal septum at the septal as well as lateral wall. An average e’ was then obtained as an average of the septal and lateral e’. By dividing the transmitral E-wave velocity by the average e’, we obtained the E/e’ ratio. LV diastolic dysfunction was then assessed in accordance to international guidelines considering four main parameters including (a) an average E/e’ of >14, (b) septal e’ velocity < 7 cm/s or lateral e’ velocity < 10 cm/s, (c) TR velocity > 2.8 m/s and (d) LA volume index (LAVI) > 34 mL/m^2^. Fulfilling 3 or more of the aforementioned criteria defined the presence of LV diastolic dysfunction [21,22,23].

### 2.3. Statistical Analysis

Continuous variables were presented as mean ± standard deviation and comparisons were performed with the Student’s *t*-test. Categorical variables were presented as percentages and frequency while comparisons were performed with the χ^2^ test (or Fisher’s exact test where appropriate). Missing data was treated as absent and excluded from the analysis. The selection of variables for multivariate analyses was decided a-priori based on previously known factors from the literature that determine functional outcome after ET. Three multivariable logistic regression models were studied, adjusting for a combination of factors including age, gender, AF, LVEF, NIHSS score on arrival, ASPECTS, success of recanalization, as well as time to reperfusion was employed. Results presented as odds ratios (OR) with 95% confidence intervals (CI). Ordinal functional outcomes, assessed as ordinal mRS 0 to 6, between those with elevated E/e’ ratio ≥ 15 and without were compared with ordinal shift analysis—multivariate ordinal logistic regression adjusting for the same variables as the final multivariable regression model and was presented as OR with 95% CI and *p* value. The primary outcome was defined as functional independence (mRS score of 0–2 at 3 months), while secondary outcomes included in-hospital mortality, successful reperfusion and symptomatic ICH. All statistical tests were performed using IBM SPSS version 26 (Armonk, NY, USA: IBM Corp.) and Stata Statistical Software: Release 17. (College Station, TX, USA: StataCorp LLC) where *p*-value of <0.05 was considered statistically significant.

## 3. Results

### 3.1. Study Characteristics

The study included 261 patients with LVO AIS who underwent ET. This comprised 118 females and 143 males with a mean age of 65.0 ± 13.9 years.

Table 1 illustrates the baseline characteristics of the study population. There were 82 (31.4%) patients with LVDD. This subgroup had a greater proportion of females (61.0% vs. 38.0%, *p* = 0.001) with a higher prevalence of comorbidities including diabetes (40.7% vs. 21.5%, *p* = 0.001), ischaemic heart disease (37.0% vs. 18.6%, *p* = 0.001), previous heart failure (23.3% vs. 7.4%, *p* = 0.001), previous strokes or TIA (24.7% vs. 10.7%, *p* = 0.004) and atrial fibrillation (59.8% vs. 44.1%, *p* = 0.019).

Table 2 shows the echocardiographic findings in the study. Patients with LVDD had larger indexed mean left ventricular (LV) dimensions including indexed EDV (72.7 ± 30.0 mL vs. 60.1 ± 18.3 mL, *p <* 0.001), indexed ESV (35.9 ± 26.8 mL vs. 22.6 ± 13.9 mL, *p <* 0.001) as well as mass index (LVMI) (117.4 g/m^2^ vs. 99.0 g/m^2^, *p* = 0.022). A greater proportion of patients with LVDD also had concurrent LV dysfunction (LVEF < 50%) (43.9% vs. 11.7%, *p <* 0.001).

Patients with LVDD suffered strokes of greater severity as represented with higher NIHSS scores (21 vs. 19, *p* = 0.048) and lower ASPECTS (8 vs. 9, *p* = 0.042) on presentation. A greater proportion of the strokes in this population were also cardioembolic in nature (62.2% vs. 47.1%, *p* = 0.015). The sites of occlusion were similar in patients with or without LVDD (*p* = 0.819).

### 3.2. Univariate and Multivariable Analysis

Between patients with or without LVDD, the proportion of successful reperfusion (78.0% vs. 79.3%, *p* = 0.870), time from stroke onset to groin puncture (282.5 ± 189.0 min vs. 320.7 ± 267.3, *p* = 0.090) and time from onset to reperfusion (363.0 ± 225.3 min vs. 391.1 ± 312.5 min, *p* = 0.591) were similar. More patients with LVDD did not achieve functional independence at 3 months (70.7% vs. 51.8%, *p* = 0.002) and a greater proportion met with in-hospital mortality (18.3% vs. 5.6%, *p* = 0.002), as shown in Table 3. Results were similar in a sub-group analysis of patients with successful reperfusion (eTICI ≥ 2b), with a lower incidence of functional independence at 3 months (34.9% vs. 65.1%, *p* = 0.004) and a higher incidence of in-hospital mortality (14.8% vs. 4.6%, *p* = 0.027) among patients with LVDD. We moved on to multivariable analysis and corrected for a combination of a priori determined covariates such as age, gender, AF, LVEF, NIHSS score, ASPECTS, time to reperfusion as well as whether recanalization was successfully achieved, in 3 separate permutations as shown in Table 3. LVDD was associated with poor long-term recovery outcomes (OR 2.18, 95% CI 1.04–4.54, *p* = 0.038) but was not associated with adverse short-term outcomes (OR 2.18, 95% CI 0.60–7.99, *p* = 0.240), as shown in Table 3. Of note, ASPECTS and incidents of unsuccessful recanalization were also significantly associated with mortality and poor functional outcome in the final model, retaining a *p* value of <0.01.

**Figure 1:**
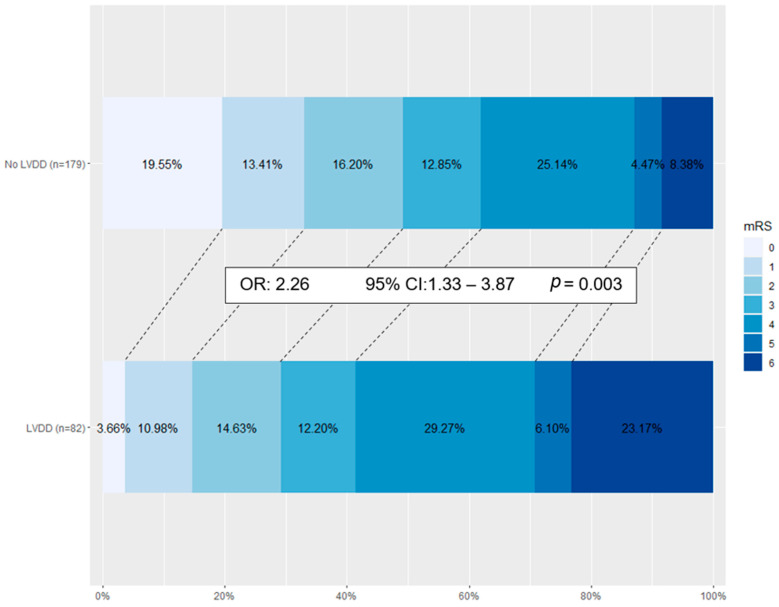
Ordinal shift regression analysis of 90-day modified Rankin Score comparing anterior large vessel occlusion stroke patients with normal and LVDD adjusted for age, gender, AF, LVEF, NIHSS, ASPECTS score, recanalization status, and time to reperfusion. AF, atrial fibrillation; ASPECTS, Alberta stroke programme early CT score; LVEF, left ventricular ejection fraction; NIHSS, National Institutes of Health Stroke Scale.

On ordinal shift analysis, LVDD was associated with an unfavourable shift in the mRS outcomes (OR 2.26, 95% CI 1.33–3.87, *p* = 0.003), as seen in Figure 1. This remained significant after adjusting for age, gender, AF, LVEF, NIHSS, ASPECTS score, recanalization status, time to reperfusion as demonstrated in the final multivariate regression model.

## 4. Discussion

Our study demonstrates a relationship between LVDD and risk of mortality and morbidity among LVO AIS patients treated with ET. Our findings may allow for better risk stratification in LVO AIS patients who undergo ET and also offer potential avenues to optimize post stroke care.

While the increasing use of ET among LVO AIS patients has significantly improved the functional outcomes, mortality and morbidity still remain high [1,2,4]. Cardiac comorbidities are common in stroke patients and LVO stroke patients are no exception [24,25]. Ischaemic cardiomyopathy was present in 16.5% of the patients in the Randomized Trial of Revascularization with Solitaire FR Device versus Best Medical Therapy in the Treatment of Acute Stroke Due to Anterior Circulation Large Vessel Occlusion Presenting within Eight Hours of Symptom Onset (REVASCAT) trial, while 14.6% had heart failure in the Randomized assessment of rapid endovascular treatment of ischaemic stroke (ESCAPE) trial [26,27]. This may be explained by the shared pathophysiology between LVO strokes and ischaemic heart disease [24]. Studies have also shown that cardiac diseases, in particular LVSD, are associated with higher rates of in-hospital mortality and poorer functional outcomes [6,28]. This effect remains significant even after adjusting comorbidities such as age, and previous ischaemic heart disease as well as the efficacy and rate of ET. Despite this, the recognition of the contribution of cardiac diseases to the outcomes of stroke remains under-documented. Most contemporary trials and registries studying LVO AIS and ET do not formally report baseline data on cardiac diseases or do so with significant heterogeneity [29,30,31]. Even for the cardiac data collected, most focused on systolic dysfunction while other indices of diastolic dysfunction and filling pressures mostly remain neglected.

In our cohort of LVO AIS patients who underwent ET, LVDD was associated with poorer functional recovery at 3 months, despite largely similar rates of reperfusion. However, in the short term, the association between LVDD and in-hospital mortality was not significant after adjusting for other variables, most notably the ASPECTS and NIHSS score. Perhaps, patients with LVDD tended to suffer larger strokes as evidenced by a poorer ASPECTS and NIHSS score on presentation. This can directly lead to a higher risk of short-term mortality due to increased risk of haemorrhagic transformation or fatal brain oedema. Furthermore, patients with LVDD and cardiac embolism suffer from more severe strokes than other ischaemic stroke subtypes [32]. LVDD has been shown to correlate well with atrial remodelling as represented by LA volumes and strain [33,34], which can predispose to regional stasis, a hypercoagulable state, and likely undiagnosed AF, leading to cerebral embolization.

In terms of functional recovery, LVDD was associated with poorer functional recovery at 3 months. This effect remained strong despite correcting for age, recanalization status, the severity of the stroke as well as LVEF. Studies have shown that early intensive mobilization with supervised aerobic training can improve functional recovery after stroke as compared to low intensity of exercise [35,36]. Thus, an LVDD even in asymptomatic cases, may indicated poorer cardiac functional reserves and thereby impede exercise capacity and neurorehabilitation and thereby impede functional recovery.

Previous studies have also shown that LV filling pressure is a predictor of adverse outcome in patients with acute myocardial infarction. Tai et al. reported that short-term adverse composite endpoint events were predicted by E/e’ in patients with ST elevation myocardial-infarction (STEMI) [9]. It is therefore interesting to note that even in patients with AIS treated with ET, E/e’ could also portend poorer outcomes through cardiac events.

To the best of our knowledge, this study is the first to examine the relationship between left ventricular filling pressures and diastolic dysfunction in LVO AIS patients after ET. However, the study was a retrospective in which where patients’ baseline characteristics and endovascular techniques were not controlled, and this heterogeneity may confound clinical outcomes. Furthermore, the timing of the TTE was also not standardized. While some patients underwent TTE, prior to ET, it was performed after ET among others. Measurements of echocardiographic indices such as LVEF and even more significantly LVDD could have been affected by the timing TTE since factors such as fluid status, permissive hypertension and sympathetic activation could have affected the haemodynamic and filling pressures in these patients. As such, ideally, a repeat echocardiogram would have been done at a later stage to re-evaluate the filling pressures for more accurate analysis. Lastly, the patients were not followed for their cardiac functional status and parameters such as the New York Heart Association (NYHA) class were not established, limiting our ability to evaluate whether symptoms of heart failure had contributed to the poorer functional outcomes observed at 3 months in the group with elevated LVDD.

## 5. Conclusions

Our study shows that attention should be paid to cardiac indices such as filling pressures and markers of diastolic function in addition to the conventional parameters such as ejection fraction and systolic function among patients with LVO AIS. In addition to providing information about the underlying aetiology, echocardiographic evaluation can offer valuable insight towards prognosis, functional recovery and outcomes after an acute stroke.

## Figures and Tables

**Table 1:** Baseline characteristics of the study population.

Variable	All (n = 261)	No LVDD (n = 179)	LVDD (n = 82)	Mean Difference	*p*-Value
Clinical Characteristics					
Age (years)	65.0 ± 13.9	63.7 ± 13.5	67.7 ± 14.3	1.02 (1.00–1.04)	0.033
Female (n, %)	118 (45.2)	68 (38.0)	50 (61.0)	2.55 (1.49–4.36)	0.001
Ethnicity (n, %)					0.589
Chinese	174 (66.7)	125 (71.0)	49 (60.5)		
Malay	50 (19.2)	26 (14.8)	24 (29.6)		
Indian	23 (8.8)	18 (10.2)	5 (6.2)		
Others	24 (5.3)	7 (4.0)	3 (3.7)		
Systolic blood pressure (mmHg)	152 ± 28	150 ± 27	156 ± 29	1.01 (0.99–1.02)	0.115
Diastolic blood pressure (mmHg)	85 ± 20	84 ± 21	87 ± 20	1.00 (0.99–1.02)	0.46
Smoker	56 (21.5)	38 (22.4)	18 (22.2)	0.99 (0.53–1.88)	0.981
Co-morbidities (n, %)					
Hypertension	188 (72.0)	118 (68.6)	70 (86.4)	2.91 (1.43–5.94)	0.002
Diabetes mellitus	70 (26.8)	37 (21.5)	33 (40.7)	2.51 (1.41–4.45)	0.001
Dyslipidaemia	138 (52.9)	87 (50.6)	51 (63.0)	1.66 (0.97–2.85)	0.065
Ischaemic heart disease	62 (23.8)	32 (18.6)	30 (37.0)	2.57 (1.42–4.65)	0.001
Heart failure	29 (12.3)	12 (7.4)	17 (23.3)	3.80 (1.70–8.45)	0.001
Previous stroke/transient ischaemic attack	38 (14.6)	18 (10.7)	20 (24.7)	2.75 (1.36–5.55)	0.004
Atrial Fibrillation	128 (49.0)	79 (44.1)	49 (59.8)	1.88 (1.11–3.20)	0.019
TOAST classification					0.015
Large vessel atherosclerosis	46 (17.6)	37 (20.7)	9 (11.0)		
Cardioembolism	123 (47.1)	72 (40.2)	51 (62.2)		
Small vessel occlusion	-	-	-		
Undetermined aetiology	90 (34.4)	68 (38.0)	22 (26.9)		
Other determined aetiology	2 (0.8)	2 (1.0)	0 (0)		
Site of Occlusion					0.819
Basilar	38 (14.6)	25 (14.0)	13 (15.9)		
M1–MCA	143 (54.8)	102 (57.0)	41 (50.0)		
M2–MCA	32 (12.3)	18 (10.1)	12 (14.6)		
Carotid	37 (13.2)	25 (14.0)	12 (14.6)		
Tandem ICA-MCA occlusion	11 (4.2)	8 (4.5)	3 (3.7)		
Investigation Findings/Procedure					
NIHSS on arrival (IQR)	20 (14–24)	19 (13–23)	21 (18–25)	0.94 (0.89–0.99)	0.048
ASPECTS score (IQR)	9 (7–9)	9 (7–9)	8 (6–9)	0.85 (0.72–0.99)	0.042
Time to Puncture (min)	310.7 ± 249.5	320.7 ± 267.3	282.5 ± 189.0	1.00 (0.99–1.00)	0.09
Time to Reperfusion (min)	382.6 ± 288.4	391.1 ± 312.5	363.0 ± 225.3	1.00 (0.99–1.00)	0.591
Outcomes					
Successful reperfusion (mTICI ≥ 2b)	206 (78.9%)	142 (79.3%)	64 (78.0%)	0.93 (0.49–1.75)	0.87
Symptomatic ICH	21 (8.0%)	12 (6.7%)	9 (11.0%)	1.72 (0.69–4.25)	0.326
In-hospital mortality	25 (9.6%)	10 (5.6%)	15 (18.3%)	3.78 (1.62–8.84)	0.002
Poor functional recovery at 3 months (MRS > 2)	149 (57.1%)	91 (51.8%)	58 (70.7%)	2.34 (1.34–4.09)	0.002

ASPECTS, Alberta stroke programme early CT score; ICA, internal carotid artery; MCA, middle cerebral artery; MRS, modified Rankin scale; mTICI, modified treatment in cerebral infarction score; NIHSS, National Institutes of Health Stroke Scale.

**Table 2:** Echocardiographic Data for the Study Population.

Variable	All (n = 261)	No LVDD (n = 179)	LVDD (n = 82)	Mean Difference	*p*-Value
Echocardiographic measurements					
Diastolic interventricular septal thickness (mm)	10.7 ± 2.6	10.5 ± 2.4	11.0 ± 3.0	1.07 (0.97–1.18)	0.473
Diastolic posterior wall thickness (mm)	10.0 ± 2.0	9.8 ± 2.0	10.3 ± 1.9	1.10 (0.96–1.26)	0.892
Left ventricular end diastolic volume index (mL/m^2^)	64.0 ± 23.3	60.1 ± 18.3	72.7 ± 30.0	1.02 (1.01–1.04)	<0.001
Left ventricular end systolic volume index (mL/m^2^)	26.8 ± 19.9	22.6 ± 13.9	35.9 ± 26.8	1.04 (1.02–1.05)	<0.001
Left ventricular mass index (g/m^2^)	104.8 ± 33.2	99.0 ± 29.2	117.4 ± 37.8	1.02 (1.01–1.03)	0.018
Left ventricular contractile function					
Left ventricular ejection fraction (%)	56.2 ± 13.2	59.2 ± 10.3	49.7 ± 16.2	0.95 (0.93–0.97)	<0.001
LV dysfunction (LVEF < 50%)	57 (21.8)	21 (11.7)	36 (43.9)	5.88 (3.14–11.06)	<0.001
Presence of regional wall motion abnormality	89 (34.2)	48 (27.0)	41 (50.0)	2.71 (1.57–4.67)	0.042
Peak systolic velocity, s’ (cm/s)	7.6 ± 3.5	8.3 ± 2.7	6.1 ± 4.5	0.69 (0.60–0.79)	0.001
Diastolic Function					
Septal E/A ratio	0.92 ± 0.63	0.93 ± 0.67	0.87 ± 0.46	1.05 (0.64–1.72)	0.61
Trans-mitral E/A ratio	1.15 ± 0.76	1.18 ± 0.79	1.06 ± 0.66	0.84 (0.52–1.35)	0.38
Mitral valve deceleration time (ms)	182 ± 59	180 ± 46	191 ± 87	1.01 (0.99–1.01)	0.062
Valvular Heart Disease					
At least moderate mitral regurgitation	13 (5.0)	6 (3.4)	7 (8.5)	0.37 (0.12–1.14)	0.074
At least moderate aortic stenosis	26 (10.0)	15 (8.4)	11 (13.4)	0.59 (0.26–1.35)	0.265
At least moderate tricuspid regurgitation	4 (1.5)	2 (1.1)	2 (2.4)	0.45 (0.06–3.27)	0.421

**Table 3:** Risk of poor outcomes in relation to LVDD by multivariable analysis.

	Mortality			Poor Functional Recovery at 3 Months		
	OR (95% CI)	*p*-Value	Chi Square	OR (95% CI)	*p*-Value	Chi Square
Model 1/LVDD adjusted for Age, Gender, AF, LVEF	2.83 (1.06–7.60)	0.039	ꭓ^2^ = 16.528 *p* = 0.006	1.95 (1.02–3.71)	0.044	ꭓ^2^ = 27.187 *p* < 0.001
Model 2/LVDD adjusted for Age, Gender, AF, LVEF, NIHSS, ASPECTS score	1.97 (0.56–6.95)	0.290	ꭓ^2^ = 21.518 *p* = 0.003	2.15 (1.05–4.44)	0.038	ꭓ^2^ = 30.206 *p* < 0.001
Model 3/LVDD adjusted for Age, Gender, AF, LVEF, NIHSS, ASPECTS score, recanalization status, time to reperfusion	2.18 (0.60–7.99)	0.240	ꭓ^2^ = 26.825 *p* = 0.002	2.18 (1.04–4.54)	0.038	ꭓ^2^ = 33.126 *p <* 0.001

AF, atrial fibrillation; ASPECTS, Alberta stroke programme early CT score; LVDD, left ventricular diastolic dysfunction; LVEF, left ventricular ejection fraction, NIHSS, National Institutes of Health Stroke Scale.

## Data Availability

Data used for this article are available from the corresponding author on reasonable request.

## Supplementary Materials

The following supporting information can be downloaded at https://www.mdpi.com/article/10.3390/jcdd11030087/s1. Supporting information on the definitions of the NIHSS scale, ASPECTS score, modified Rankin Scale and TOAST classification are available in the Supplement. References [37,38,39,40] are cited in the supplementary materials.
