# Supplementary material for: Left Ventricular Diastolic Dysfunction Is Associated with Poor Functional Outcomes after Endovascular Thrombectomy
Source: J Cardiovasc Dev Dis. 2024 Mar 5;11(3):87. doi: 10.3390/jcdd11030087 (PMC10971731; doi:10.3390/jcdd11030087)
Supplement: Supplementary file 1 [file jcdd-11-00087-s001.zip › jcdd-2847807-supplementary.pdf]

Supplement:

The National Institutes of Health Stroke Scale (NIHSS) is a 15-item neurological examination stroke scale used to evaluate the effect of a stroke. It assesses the patient based on the levels of consciousness, language, neglect, visual-field loss, extraocular movement, motor strength, ataxia, dysarthria, and sensory loss. It is designed to be a simple and reproducible tool that can be administered in less than 10 minutes. It is one of the most commonly used stroke assessment tools today(1).

The Alberta stroke program early CT score (ASPECTS) 1 is a 10-point quantitative topographic CT scan score used for middle cerebral artery (MCA) stroke patients. It is used to determine the extent of cerebral territory that the ischemic stroke has affected. Segmental estimation of the middle cerebral artery (MCA) vascular territory is made, and 1 point is deducted from the initial score of 10 for every region involved. An ASPECTS score less than or equal to 7 predicts a worse functional outcome at 3 months as well as symptomatic haemorrhage(2).

The modified Rankin Scale (mRS) is a commonly used scale for measuring the degree of disability or dependence in the daily activities of people who have suffered a stroke or other causes of neurological disability. It has become the most widely used clinical outcome measure for stroke clinical trials (3, 4).

The scale runs from 0–6, running from perfect health without symptoms to death. The exact breakdown would be as follows:

- 0 - No symptoms.
- 1 - No significant disability. Able to carry out all usual activities, despite some symptoms.
- 2 - Slight disability. Able to look after own affairs without assistance, but unable to carry out all previous activities.
- 3 - Moderate disability. Requires some help, but able to walk unassisted.
- 4 - Moderately severe disability. Unable to attend to own bodily needs without assistance, and unable to walk unassisted.
- 5 - Severe disability. Requires constant nursing care and attention, bedridden, incontinent.
- 6 - Dead.

The TOAST (Trial of ORG 10172 in Acute Stroke Treatment) classification is a system for classifying ischemic stroke subtypes based on etiology. It comprises of five major subtypes: large artery atherosclerosis, cardioembolism, small artery occlusion, stroke of other determined cause, and stroke of undetermined cause (5).

All of these tools are readily available in the public domain and free web calculators are available.

References:

1. Brott T, Adams HP, Jr., Olinger CP, Marler JR, Barsan WG, Biller J, et al. Measurements of acute cerebral infarction: a clinical examination scale. Stroke. 1989;20(7):864-70.
2. Barber PA, Demchuk AM, Zhang J, Buchan AM. Validity and reliability of a quantitative computed tomography score in predicting outcome of hyperacute stroke before thrombolytic therapy. ASPECTS Study Group. Alberta Stroke Programme Early CT Score. Lancet. 2000;355(9216):1670-4.
3. Rankin J. Cerebral vascular accidents in patients over the age of 60. II. Prognosis. Scott Med J. 1957;2(5):200-15.
4. Banks JL, Marotta CA. Outcomes Validity and Reliability of the Modified Rankin Scale: Implications for Stroke Clinical Trials. Stroke. 2007;38(3):1091-6.

5. Adams HP, Jr., Bendixen BH, Kappelle LJ, Biller J, Love BB, Gordon DL, et al. Classification of subtype of acute ischemic stroke. Definitions for use in a multicenter clinical trial. TOAST. Trial of Org 10172 in Acute Stroke Treatment. Stroke. 1993;24(1):35-41.
